# Different Morphologies of *Leishmania major* Amastigotes with No Molecular Diversity in a Neglected Endemic Area of Zoonotic Cutaneous Leishmaniasis in Iran

**DOI:** 10.7508/ibj.2015.03.004

**Published:** 2015-07

**Authors:** Adel Spotin, Soheila Rouhani, Parnazsadat Ghaemmaghami, Ali Haghighi, Mohammad Reza Zolfaghari, Aref Amirkhani, Mahin Farahmand, Ali Bordbar, Parviz Parvizi

**Affiliations:** 1 *Molecular Systematics Laboratory, Dept. of Parasitology, Pasteur Institute of Iran, Tehran, Iran; *; 2 *Dept. of Parasitology, Medical Faculty, Shahid Beheshti University of Medical Sciences, Tehran, Iran;*; 3 *Dept. of Parasitology, Medical Faculty, Tabriz University of Medical Sciences, Tabriz, Iran; *; 4 *Dept.of Microbiology, Qom Branch, Islamic Azad University, Qom, Iran; *; 5 *Dept. of Epidemiology, Medical Sciences of Tehran branch, Islamic Azad University, Tehran, Iran*

**Keywords:** *Leishmania major*, Nuclear gene, Mitochondrial gene, Amastigote shapes, Iran

## Abstract

**Background:**

Molecular diversity of *Leishmania major* and its morphological changes have become a controversial issue among researchers. Some aspects of polymorphic shapes of amastigotes in clinical manifestations along with molecular variation were evaluated among suspected patients of some exceptional zoonotic cutaneous leishmaniasis locations in Northern Khuzestan, Southwestern Iran.

**Methods:**

Suspected patients (n = 165) were sampled in zoonotic cutaneous leishmaniasis foci over two consecutive years during 2012-2014. Prepared smears were stained, scaled and measured by ocular micrometer. DNA was extracted from smears; ITS-rDNA and Cytochrome *b* (Cyt *b*) markers were amplified, and PCR products were digested by *B*suR1 restriction enzyme. Then the RFLP and sequencing were employed.

**Results:**

Only *L. major* was identified in patients containing regular amastigotes' shapes (oval or round) with a size of 2-4 µm in each of classical wet, dry, mixed lesions. Meanwhile, irregular shapes (spindle, pear, or cigarette) were observed separately in non-classical wet lesions with more than 4 µm. Interestingly, a few amastigotes with an external flagellum were observed in some lesions. All sequenced ITS-rDNA and Cyt *b* genes of *L. major* did not show any molecular variation (χ ^2^ *P* > 0.05), including only one common haplotype (GenBank access no. EF413075).

**Conclusion:**

Findings proved that unlike other endemic foci, there is not a meaningful correlation between phenotypic and genotypic features of *L. major *isolates. This study is considered as the first comprehensive report to incriminate morphometric shapes of *L. major* amastigotes, which enhances our knowledge concerning their relevance with various clinical appearances and genotypic traits.

## INTRODUCTION

Leishmaniasis is a group of neglected tropical diseases with various clinical manifestations in Iran and the world [1]. *Leishmania *parasites, the causative agents of leishmaniasis (Kinetoplastida: Trypanosomatidae), can be transmitted from reservoir hosts to human by sandflies’ bite [2-4].

In Iran, three epidemiologically important forms of *Leishmania* parasites have been reported: zoonotic cutaneous leishmaniasis (ZCL), anthroponotic cutaneous leishmaniasis, and zoonotic visceral leishmaniasis [5-7].

**Fig. 1:**
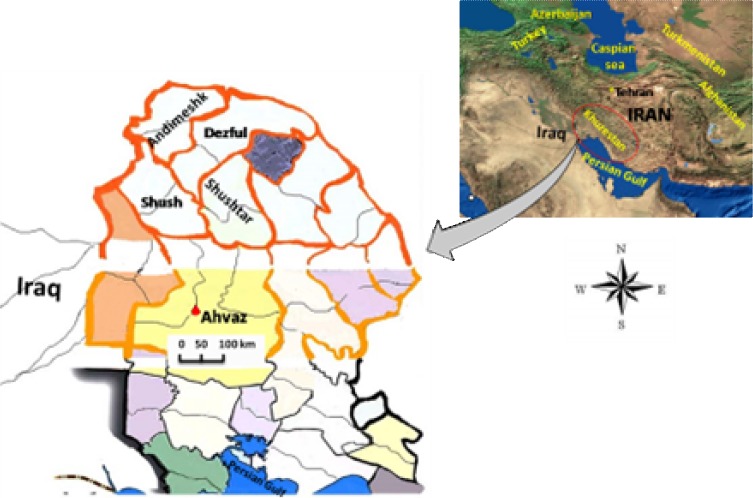
Studied locations of suspected patients in the foci of zoonotic cutaneous leishmaniasis in Northern Khuzestan (Shush, Shushtar, Dezful, and Andimeshk).

Among 17 out of 31 provinces of Iran, about 90% of reported leishmaniasis belongs to ZCL with a large geographical distribution [7, 8]. Northern Khuzestan, located in Southwestern Iran, is one of the neglected endemic areas of ZCL with high infection rate, and it has a common border with Iraq, where at least three *Leishmania *species (*L. major*, *L. tropica*, and *L. infantum*) have been confirmed in this region [9]. In this neglected area bordering with Iraq, *Leishmania major* is well known as the causative agent of cutaneous leishmaniasis. In addition, the sandfly of* Phlebotomus papatasi* and also the rodents of *Rhombomys opimus*, *Meriones libycus*, *Meriones hurrianae, Tatera indica*, and *Nesokia indica* have been introduced as proven vectors and reservoir hosts of ZCL in different parts of Iran [2, 10-13]. High prevalence rates of leishmaniasis have drawn many interests of researchers for more investigation in this region. Moreover, transporting and keeping the samples are raised to problems because of highly temperature and humidity of the weather and for impassable areas of ZCL foci. Also, some leishmaniasis regions are situated on the Iran-Iraq border, where it is not safe for sampling. Because of such limitations, Khuzestan Province remains with no comprehensive studies on leishmaniasis [9]. After the Iraq-Iran imposed war (1980-1988), the reconstructions and building settlements in new areas have induced some changes in the ecology of the reservoir hosts, vector and parasite in three important regions in north, center, and south of Khuzestan Province. One of the critical under-attacked regions was Northern Khuzestan, where Shush, Dezful, Andimeshk, Shushtar, and some neglected rural areas are situated in this district with high *Leishmania* infection rate (Fig. 1). Therefore, we designed a research project on all aspects of *Leishmania *parasites including detections, isolations, and identifications of *Leishmania *parasites in humans, mammalian reservoir hosts, and field-caught sandﬂies with the aid of local authorities from each relevant public health service. In this investigation, both conventional and molecular methods were employed to identify the different characters of* Leishmania* parasites to get the better knowledge of *Leishmania* density in different locations. Also, both nuclear ribosomal internal transcribed spacer (ITS-rDNA in nucleus) and Cytochrome *b *(Cyt *b* in mitochondrial) genes are applied to understand any relationships between amastigotes' shapes in different clinical forms and molecular diversity range of *Leishmania* parasites.

## MATERIALS AND METHODS

**Fig. 2:**
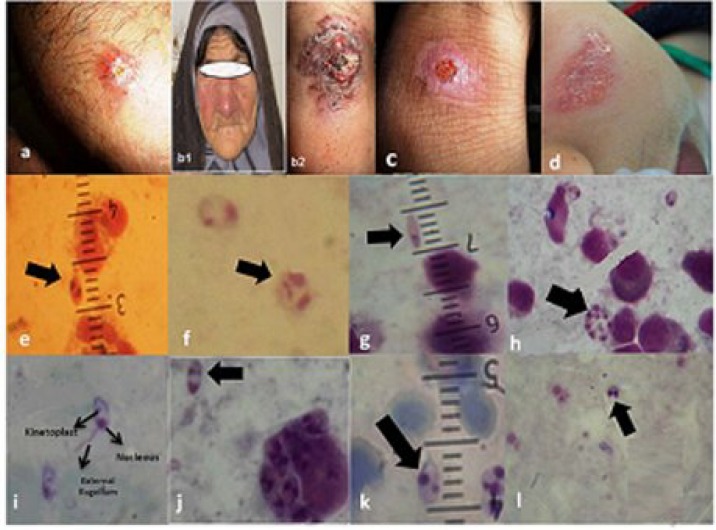
Clinical appearance of cutaneous leishmaniasis cases in four districts of Northern Khuzestan Province. (a) Mixed lesion; isolated from Shush, (b1) Non-classical wet lesion, hyperkeratotic shape: isolated from Shushtar; (b2) Erysipelas from (c) classical wet lesion: volcanic shape, isolated from Andimeshk; (d) Dry lesion,; isolated from Dezful. Observed amastigote shapes in one lesion type (1000×): (e) spindle, (f) round, (g) cigarette, (h) rosette form, (i) paramastigote form, (j) oval, (k) pear shape, (l) binary form.

***Locations, morphological identification, and animal inoculation.*** In north of Khuzestan Province (Southwestern Iran), four geographical locations (Shush, Dezful, Shushtar, and Andimeshk) were selected within the ZCL foci. These areas are placed between 32°7′ N to 32°10′N and 48°20′ E to 48°31′ E consisting of 22 villages and 6 districts. They are also situated mostly on Iran-Iraq borders within 10,566 km^2^ and 97-150 meters above sea level with high temperature (above 50ºC ) and humidity climates (95% in summer) (Fig. 1). Sampling was carried out from April 2012 to January 2014; the smears were prepared from the active lesions of suspected patients in all villages of four locations. The personal information of each suspected patient was recorded in a separate sheet. The recorded information included age, sex, duration of lesion, number and type of lesion, ulcers’ position, patient's travelling to endemic ZCL regions, and also medication consumption [12, 14]. The samples of suspected patients were smeared on two microscopic slides, air dried, fixed with methanol and stained by Giemsa. All collected smears were examined under a light microscope with high magnification (1,000×) [15]. Different sizes and shapes of amastigotes from each infected lesion were accurately tested (30 minutes per slide) by Dino Capture 2.0 software and a light microscope equipped with an ocular micrometer (1,000×, which was previously calculated by dividing ocular micrometer to stage micrometer (100 × objective = 1 µm per unit space) (Fig. 2). The positive smears from each patient were scored for amastigote density from +1 to +6 [15]. Serous of some samples were subcutaneously inoculated into the base tail of the BALB/C and examined weekly with intent to examine the appearance of lesion at the position of injection for six months. Some sera from the suspected patients were cultured in Novy–MacNeal–Nicolle medium and those with active lesion were incubated at 22^o^C for six weeks. Weekly sub-cultured samples were checked regularly to monitor the growth and the presence of promastigotes. Experiments involving animals or human material were reviewed by the appropriate review board/ethics committee of Pasteur Institute of Iran (No., 605).

***Extraction of total genomic DNA.*** All smears from Giemsa-stained slides were washed with ethanol and covered by 300 µl lysis buffer. In our modified DNA extraction method, tubes containing lysis buffer were incubated at 56°C for 24 hours instead of using proteinase K. The smears were removed completely and transferred to a 1.5-ml reaction tube. Then, the extraction of the genomic DNA of each Giemsa-stained slide from the suspected patient and any parasite within was followed by the modified method of Bordbar and Parvizi [14].

**Fig. 3:**
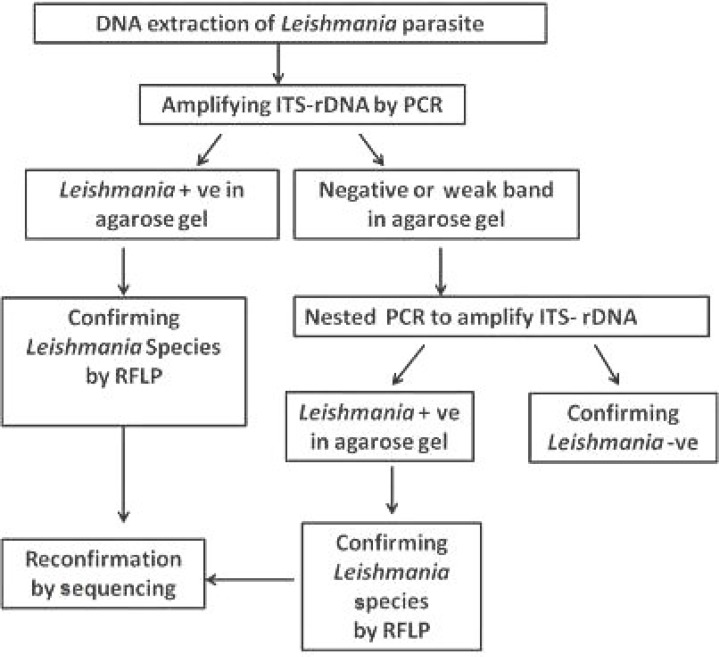
*In silico* prediction of ITS-rDNA restriction fragments of the ITS1-5.8S rRNA–ITS2 amplification products in the *Leishmania* species based on GenBank sequences, Negative (-ve), Positive (+ve).

***PCR amplification of*** ***ITS-rDNA and Cytochrome b genes*** ***.*** The nested PCR was employed to screen *Leishmania *infection in suspected patients using ITS-rDNA and Cyt* b *gene amplification. The primer pairs were IR1 and IR2 for the first step and ITS1F and ITS2R4 for the nested PCR. The details of PCR protocol were the same as reported before [6, 16]. Double distilled water was used as a negative control and DNA from *L. major *as a positive control for each batch of PCR. The molecular analyses are briefly shown in Figure 3.

**Fig. 4:**
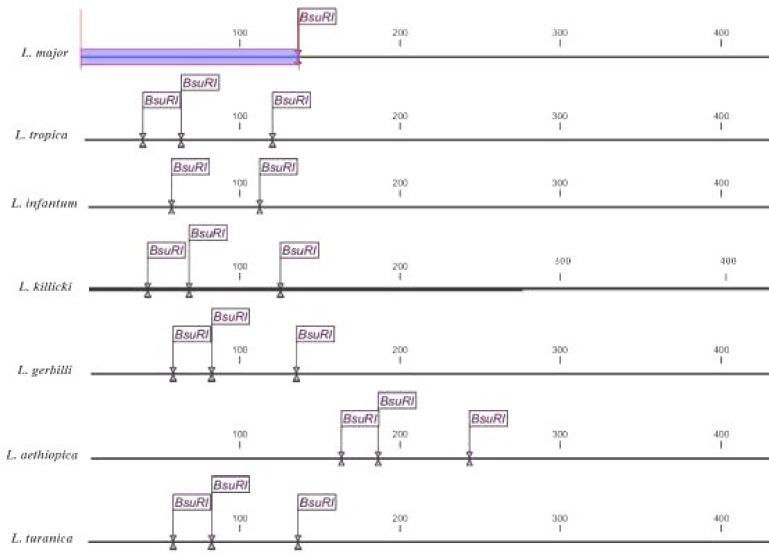
Proposed scheme of the PCR diagnosis procedure for characterization of *Leishmania* parasites in this study

**Fig. 5:**
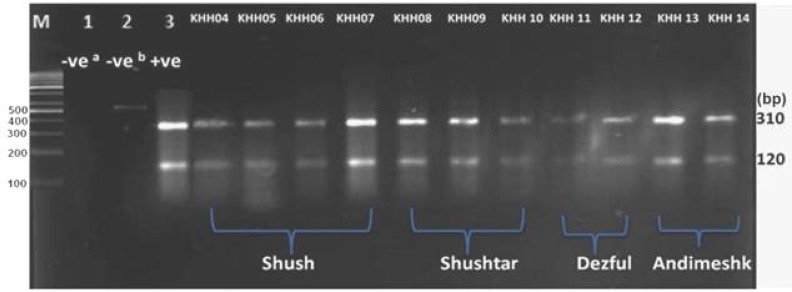
Results of PCR-RFLP in isolates from Northern Khuzestan. Lane 1, -ve^a^: negative control containing *B*suR1 without PCR product; Lane 2, -ve^b^: negative control containing PCR product without *B*suR1; Lane 3, +ve: positive control for* L. major*, and KHH04-07, *L. major*: isolated from Shush; KHH08-10, *L. major*: isolated from Shushtar, KHH11 and 12, *L. major*: isolated from Dezful; KHH13 and 14, *L. major*: isolated from Andimeshk. M, 100 bp size marker

***RFLP*** ***for ITS-rDNA gene by in silico analyses*** **. **The sequences of ITS-rDNA gene of Old World *Leishmania* (*L. major*: EF413078.1,* L. tropica*: KC540906*, L. infantum*: EU330402*, L. turanica*: EF413079, and *L. gerbilli*: AJ300486) in GenBank were saved for sequencing analyses. An appropriate restriction enzyme was utilized for various species of *Leishmania Bsu*RI (*Hae*III) with the cut site GG↓CC for the digestion of *Leishmania *PCR product using CLC DNA Workbench 5.2 software (CLC bio A/S, Aarhus, Denmark) (Fig. 4). After the digestion of PCR product, we analyzed the fragments using electrophoresis on agarose gel 3% containing ethidium bromide and ladder 100 bp (Fermentas, UK) (Fig. 5).

***DNA sequencing for ITS-rDNA and Cytochrome b. ***The data were directly analyzed from PCR products of ITS-rDNA and Cyt* b* to identify *Leishmania* variation (strain or haplotype) in infected patients. A number of PCR products amplified from ITS-rDNA gene of *Leishmania* parasites from suspected patients were sequenced to confirm the results of RFLP and firmly to identify *Leishmania* species. Both directions of our sequences were aligned and edited using Sequencher^TM^v. 4.1.4 Software for PC (Gene Codes Corporation, USA). MEGA v5.05 software was used for phylogenetic analysis and compared with some GenBank sequences of all regional species in case of homology and similarity [17]. In this study, a graph was made using GraphPad Prism 5 (Graphpad Software, California, USA).

## RESULTS

***Morphometric identification of Leishmania*** ***parasites in suspected patients.*** Suspected patients (n = 165) with acute or any sign of lesion were selected and examined microscopically for *Leishmania* infections. Among them, 127 people were found to be infected with *Leishmania *parasites. All information of suspected patients is shown in Table 1.* Leishmania* infections were more in group of ages 5-10 years old (35.4%), predominantly in males (69.3%) (Table 1). It is notifying that we expected to observe only oval and round shapes of *L. major* [18, 19]. In this study, *L. major *amastigotes were seen in each lesion of patient with five polymorphic shapes, including regular shapes: round (16.5%) and oval (66.9%) (Fig. 2f and 2j) and unexpected irregular shapes: spindle (3.14%), cigarette (7%), and pear (6.3%) (Fig. 2e, 2g, and 2k) (Table 1).Various amastigote shapes of *L. major* were differentiated accurately in case of their morphometric characters and the direction of nucleus and kinetoplast. Interestingly, some exceptional amastigotes had small external flagellum (Table 1, Fig. 2i). In other prepared samples from the ulcers of suspected patients, amastigote directions were observed in rosette and binary forms (Fig. 2h and 2 l).

Generally, the wet lesions of *L. major* are principally categorized into classical (as a routine form namely volcanic shape) and non-classical lesions (herpetic form, erythematous, papulonodules, hyperkeratotic, eczematoid, zosteriform, and psoriasiform patterns). Principally, round or oval (regular) amastigote shapes were specifically visualized in each of the dry (33%), mixed (11%), or wet (mainly in classical form; volcanic: 39.4%) lesions: 83.5%, (Table 1, Fig. 2a, 2c, 2d, 2f, and 2j) while the irregular amastigote shapes (cigarette, pear, or spindle) were individually observed in each of the non-classical wet lesions: hyperkeratotic, Erysipelas, eczematoid, and pustule, (16.5%) (Fig. 2b1, 2b2, 2e, 2g, and 2k). Regarding amastigote morpho-metrics, all sizes of irregular shapes were observed with more than 4 µm (16.5%) (Fig. 2e, 2g, and 2k). Conversely, the expected sizes of round or oval amastigote shapes were ranging from 2-3 µm (31.5%) to 3-4 (51.9%) µm (Fig. 2f and 2j), including mixed, classical wet or dry lesions (Fig. 2a, 2c, and 2d, respectively).

**Fig. 6:**
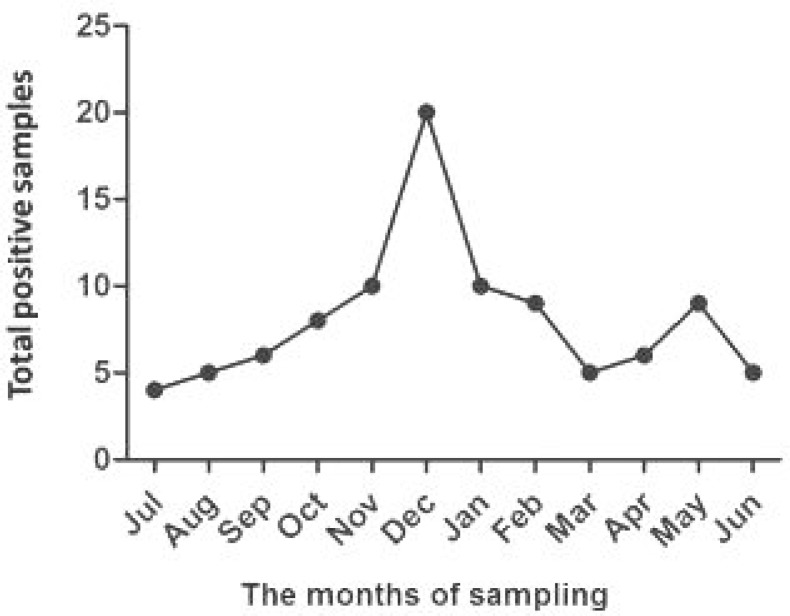
Seasonal activity of *L. major* sampled in villages of Northern Khuzestan from 2012 to 2014.

Patients’ lesions were characterized based on indifferent positions of the body. Hands (36.2%) had more *Leishmania* parasites than feet (25.2%), face and neck (14%), and other parts of the body (scapula, legs, back of body, knees, and arms) (24.4%). The majority of acute sign of lesions were found in the first month of the sandfly bite (Table 1).

The grades of +3 (24.4%) and +4 (23.6%) were more than the rest. The slides were measured by ocular micrometer and showed 2-3, 3-4, and more than 4 µm. Also, the majority of measures were 3-4 µm (51.9%) (Table 1).

The number of people who had *Leishmania* lesions in their bodies was more in Shush (48.8%) than Dezful (28.3%), Shushtar (14.1%), and Andimeshk (8.6%) (Table 2). The incidence rate of leishmaniasis was high in December (20) and low in July (4) (Fig. 6). In Khuzestan, unlike the other endemic Provinces of Iran, sandflies have two peaks of seasonal activity each year. Owing to high temperature (50ºC) and humidity (95%) in late winter (in early March) and late summer (in mid-September), we faced a sharp increasing of leishmaniasis incidence rate in December (20) and May (9) (Fig. 6).

***Species identification of Leishmania parasites using RFLP and sequencing of ITS-rDNA and Cytochrome b genes.*** Of 165 *Leishmania* samples, 135 were positive and identified by targeting ITS-rDNA and Cyt *b* genes. The *Leishmania* infections were obtained from one (41%) to five (4.7%) ulcers (Table 1). Of 135 *Leishmania* positive, 97 (72.8%) were digested by *Bsu*RI (HaeIII) and *S*sp1 and sequenced to identify *Leishmania* species, which all 97 parasites were *L. major*. Also, 38 (28.1%) out of 135 PCR products did not have enough DNA for digesting by *Bsu*RI enzyme and/or sequencing. No variation was found among 27 sequences of ITS-rDNA and Cyt *b* fragments of *L. major*. Only a common haplotype (ITS, GenBank accession no. EF413075 and Cyt *b*, GenBank accession no. AB095961) was identified, which had previously been submitted and reported from sandflies, rodents, and humans in Iran and Japan [6]. This haplotype did not have any nucleotide differentiation from other common haplotypes (GenBank accession nos. AJ300481 and AY283793) [2, 6]. The RFLP method was allowed differentiating from each species of *Leishmania* parasites unambiguously. Two fragments of 140 and 340 bp were assigned to *L. major* while the other four fragments of 30, 40, 50, and 340 bp were belonged to* L. tropica* and *L. turanica*. Likewise, three fragments of 50, 70, and 340 bp were accepted for *L. infantum* (Fig. 4). Also, the sequences of ITS-rDNA and Cyt *b* in comparison to those of GenBank had 100% similarity and homology, and only *L. major* was firmly identified (Table 2).

## DISCUSSION

In the current study,* Leishmania* parasites were obtained from suspected patients in four districts of Northern Khuzestan Province over two successive years. The only detected infection was *L. major *containing five different morphometric shapes of amastigotes (regular: oval and round, irregular: cigarette, pear, and spindle) along with different sizes of amastigotes (regular shape: 2-4 µm and irregular shape: >4 µm) in various clinical forms (wet: classical/non-classical, mixed, and dry). Nevertheless, only one common haplotype was identified in *Leishmania* parasite strains with no remarkable diversity compared to a previous report [6] (GenBank access No. EF413075).

In the present findings, revealing different morphological amastigote shapes with no molecular variation cannot be always justified because we used nuclear ITS-rDNA gene, which is usually considered as a conserved gene. However, examining Cyt *b *extra nuclear gene did not show remarkable nucleotide variation among our analyzed sequences. In addition, we expected that Cyt *b* can detect the point mutations easier than nuclear gene (ITS-rDNA) [20]. It is probable that the use of mitochondrial markers (kDNA and COII) gives rise to find nucleotide variations in different morphological amastigote shapes of *Leishmania* species [21, 22]

Spotin *et al.* [23] have recently reported the genetic features of *L. tropica* and *L. major* in center of Khuzestan. L. tropica has been shown to have more genetic diversity than L. major based on ITS-rDNA and Cyt b genes. Also Sharbatkhori *et al.* [24] has been shown that *L. major* based on ITS-rDNA gene has low diversity with only one common haplotype from sandflies species of a zoonotic cutaneous leishmaniasis in northeast of Iran. Maraghi *et al.* [25] have reported that* L. major* and *L. tropica *were isolated from 45 and four patients in Northern Khuzestan, respectively by observing DNA band in agarose gel. However, molecular analyses of *Leishmania* parasites have been rarely sequenced in this region, and there is no apparent conception of *Leishmania* species identification [9, 25, 26].

**Table 1:** The characteristics of confirmed cutaneous leishmaniasis from patients based on personal information, lesion characteristics, scaling slides, shapes and sizes of amastigotes

**Criteria**					**Skin lesion sites**					**Lesion duration (month)**				**Number of lesions**				**Lesion types**					**Grading slides**						**Morphometric characteristics**									
																														**Sizes of amastigotes (** **µm** **)**				**Shapes of amastigotes**				
**Age group (yr)**		**Sex**			**Face & Neck**	**Hand**	**Feet**	**Other**		**1**	**1-2**	**>3**		**Single**	**Double**	**Multiple**		**Wet**		**Dry**	**Mixed**		**+1**	**+2**	**+3**	**+4**	**+5**	**+6**		**2-3**	**3-4**	**>4**		**Regular**		**Irregular**		

**Age ranges**	**Total**	**F**	**M**															**C**	**NC**															**Round**	**Oval**	**Pear**	**Cigarette**	**Spindle**
<1	4	2	2		1	1	0	2		3	0	1		1	1	2		3	0	1	0		0	0	1	2	1	0		2	2	0		2	1	0	1	0
1-3	11	2	9		1	3	6	1		5	4	2		4	2	5		3	2	4	2		1	4	0	5	1	0		2	7	4		2	7	1	1	0
3-5	14	3	11		2	4	4	4		3	3	8		5	4	5		5	4	5	0		1	2	3	6	1	1		8	5	2		3	10	1	0	0
5-10	45	10	35		6	14	10	15		23	12	10		23	11	11		12	9	18	6		8	9	8	7	6	7		20	15	8		8	32	3	2	0
10-15	11	5	6		3	4	2	2		6	5	0		2	4	5		6	2	2	1		0	0	4	1	3	3		1	8	2		1	8	1	1	0
15-25	18	6	12		2	8	5	3		6	9	3		9	7	2		10	3	4	1		1	1	6	2	6	2		2	12	3		2	11	1	2	2
>25	24	11	13		3	12	5	4		11	12	1		8	10	6		11	1	8	4		1	3	9	7	4	0		5	17	2		3	16	1	2	2
Total (%)																																						
	127	39	88		18	46	32	31		57	45	25		52	39	36		50	21	42	14		12	19	31	30	22	13		40	66	21		21 (16.5)	85 (66.9)	8 (6.3)	9 (7)	4 (3.14)
		(30.7)	(69.3)		(14)	(36.2)	(25.2)	(24.4)		(44.8)	(35.4)	(19.6)		(41)	(30.7)	(28.3)		(39.4)	(16.5)	(33)	(11)		(9.4)	(15)	(24.4)	(23.6)	(17.5)	(10.2)		(31.4)	(51.9)	(16.5)		106 (83.5)		21 (16.5)		
																																	
		127			127					127				127				127					127							127				127				

Abbreviations: Sex: F=Female, M=Male; Skin lesion sites: Other; Scapula, Leg, Back, knee, arm , Lesion types: C:Classic , NC: Non-Classic, Grading slides: Positive: 1-6, 0; 0 Parasite / 1000 fields, +1: 1-10 Parasite / 1000 fields,+2: 1-10 Parasite / 100 fields,+ 3: 1-10 Parasite / 10 fields, +4: 1-10 Parasite / 1 field, +5: 10-100 Parasite / 1 field, +6:>100 Parasite /1 field**.**

**Table 2:** Detection of cutaneous leishmaniasis in suspected patients of Northern Khuzestan based on molecular analyses and locations -Ve: Negative; +Ve: Positive

** Locations**					***Leishmania*** ** +Ve** **based on** **Giemsa-stained smears**				**RFLP-PCR with ** ***B*** **suR1for ITS-rDNA gene**	
**Province**	**Districts**	**Villages**	**Altitude (m)**	**Total ** **sample examination**						
					**Microscopic** **observation**	**Molecular by ITS-rDNA and Cyt ** ***b *** **genes**		**-Ve**	***L. major*** No.	**Other Species no.**
**Northern Khuzestan**	**Shoush**	Haftape	97	10	7/10	8/10		2	5	0
		Sorkhe	97	8	7/8	6/8		1	7	0
		Aljazayer	97	14	11/14	13/14		1	9	0
		Banader	97	2	2/2	2/2		0	2	0
		Seyedrahime	97	1	1/1	1/1		0	1	0
		Montazeri	97	2	2/2	2/2		0	2	0
		San karim	97	1	1/1	0/1		0	1	0
		Akharasfalt	97	22	15/22	18/22		3	5	0
		Horr	97	6	4/6	5/6		1	3	0
		Sheykh Nader	97	4	3/4	3/4		0	3	0
		Choghazanbil	97	3	2/3	2/3		0	2	0
		Joohi	97	1	1/1	0/1		0	1	0
		Maraghiye	97	3	3/3	2/3		0	3	0
		Seyedrazi	97	4	3/4	4/4		0	3	0
	**Dezful**	Zaviehoradi	103	8	7/8	8/8		0	7	0
		Deylameofla	103	6	5/6	6/6		0	4	0
		Kheybar	103	3	3/3	3/3		0	2	0
		Beheshti	103	7	3/7	4/7		3	4	0
		Chogapahn	103	8	5/8	3/8		3	3	0
		Gavmish bad	103	2	2/2	2/2		0	2	0
		Baghcheban	103	3	3/3	3/3		0	3	0
		Hamzearoon	103	7	5/7	5/7		2	4	0
		Fazili	103	4	3/4	3/4		1	2	0
	**Shushtar**	Galegah	93	6	6/6	5/6		0	4	0
		Fallahi	93	7	6/7	6/7		1	4	0
		Shushtar	93	8	6/8	7/8		1	3	0
	**Andimeshk**									
			150	15	11/15	14/15		1	8	0
Total (%)				165	127/165 (76.9)	135/165 (81.8)		20/165 (12.2)	97/135 (72.8)	0

Though various clinical manifestations of *L. major* with different genetic diversity were found in this area, several genetic diversity was observed using mini-exon and kDNA genes in the region and elsewhere in Iran [21, 22], it is also not clear that how the authors identified the genetic diversity of *L. major* with or without a few sequences. In this study, a noticeable correlation was found among irregular amastigote shapes (16.5%) with a size of > 4 µm (16.5%) in non-classical wet lesions (16.5%) (χ^2^ test: *P* < 0.05). Likewise, a remarkable correlation was observed between the presence of regular amastigote shapes (round and oval; 83.5%) with different sizes of 2-3 and 3-4 µm (83.5%) into dry, mixed, and wet (mainly classical form; volcanic) lesions (83.5%) (χ^2^ test: *P* < 0.05). Hence, it seems that irregular morphometric shapes of amastigotes can insert the substantial effects on disfiguration of non-classical wet lesions (Fig. 2b1, b2, 2e, 2g, and 2k).

Finding *L. major* parasites with no molecular variation and various phenotypic characteristics (amastigote morphometric and clinical patterns) reveals that isolated *L. major* strains from Southwestern Iran have their own specific features. This evidence unequivocally indicates that different phenotypic features of *L. major *are not tightly associated with the molecular diversity ranges.

Regarding to several reasons, such as immune interaction of hosts with parasite, migration of individuals to non-endemic areas, the number of inoculated parasites by sandfly, long period of high temperature seasons, nutritional status of the hosts, wound contamination with inorganic ingredients as well as consuming oral steroids can distinctly influence on the formation of non-classical lesion appearances without considering noticeable impact on their genetic traits [27-29].

To our knowledge, two amastigote shapes (oval and round) should have been observed without expecting any other shapes [6, 11, 30]. Despite the finding of no molecular variation of ITS-rDNA and Cyt *b* genes of *L. major *in this report, low diversity of *L. major* and no variation of *L. turanica* were found in recent investigations from suspected patients of Turkmen Sahara in north of Iran [12, 14].

In this record, the molecular methods were a little more sensitive (81.8%) than parasitological strategies (76.9%), which we found in our previous investigations as well [6, 11]. The eminent reason of unusual lesions existence in suspected patients and deformation of regular amastigote shapes can be explained by the chemotherapy as well as effects of host immune responses or ecological conditions on parasite. These factors can lead to misdiagnosis. However, some negative molecular outcomes with positive microscopic observation should be stated by the role of inhibitory DNA agents, such as protoporphyrin, DNase, and inappropriate extraction procedures.

The ZCL infection rates were observed more in males. Because, males usually work in the farm or field and females cover their body and use Hijab owing to religion. The infection rates of 5-10 years old age ranges was higher (45/127) than other age groups because of low immunity responses of Th1 (premunition/concomitant deficiency) to* Leishmania *parasites [31].

Whereas we expected to detect *L. major *parasites in wet lesions individually, they were isolated and recognized obviously in different types of wet lesions, including classical and non-classical, dry, and mixed ulcers [21]. These surprising observations of three different ulcers can be explained by the capability of *L. major* to have tropism to clinical patterns isolated in southwestern Iran.

The dry lesions of *L. tropica* and the wet lesions of *L. major *were found by regional investigators in some parts of Khuzestan but we found only *L. major*, which look likes dry, wet, or mixed lesions [9, 26]. No significant statistical differences were observed between lesion type and the kind of *Leishmania *species (χ ^2^ test: *P* > 0.05).

Shush had higher *Leishmania* infections than other studied locations (Andimeshk, Dezful, and Shushtar) (Table 2). The gerbil burrows of rural villages in Shush district are close to residential places and flying sandflies from rodent burrows could transfer *Leishmania* parasites to human while in three other locations, gerbil burrows are far from residential places. Sandflies, rodents, and *Leishmania* parasites are three significant factors that are required to complete leishmaniasis transmission cycle. *P. papatasi *is abundant in rural villages of Shush district and rarely disperses more than 1.5 km. It is the only sandfly species that is judged as a proven vector of ZCL in Iran [32].

Interestingly, a few numbers of amastigotes with an external flagellum were observed in some lesions of the patients. These features can be a type of promastigote that is called paramastigote [33]. The external flagellum rarely exists in some species of *Leishmania* parasites in sandfly pharynx [34]. The flagellum is usually originated from the posterior region near the kinteoplast but in paramastigote form, it is originated from the anterior region near the nucleus (Fig. 2i).

One of the underlying assumption about having not genetic variation or genetic stability is the percentage of higher GC content in a genome of parasites. *Plasmodium falciparum* malaria genome has low GC content (19.2%), which is one of the remarkable reasons for instability in the genome [35]. Therefore, we inferred that observing no molecular variations in *L. major* among suspected patients in Northern Khuzestan may be associated with high GC content in *L. major* (59.7%) [36].

We can conclude that *L. major* is circulating in north of Khuzestan with high infection rates. More precisely, different morphological shapes of amastigote in various clinical manifestations with no molecular variations were detected in *L. major* by having one common haplotype sequence of ITS-rDNA and Cyt *b* genes. It should be advised to use appropriate tools for accurate and firmly identification of *Leishmania* parasites and their variations. Therefore, simultaneous evaluation of amastigote morphometric features in different clinical lesions along with choosing various molecular markers, sequencing and phylogenetic analyses must be considered in order to avoid misleading and confusing the identification of *Leishmania* parasites and molecular variations.
